# Superselective Renal Artery Embolization Management of Post-percutaneous Nephrolithotomy Hemorrhage and Its Methods

**DOI:** 10.3389/fsurg.2020.582261

**Published:** 2020-11-26

**Authors:** Xiangjun Dong, Yanqiao Ren, Ping Han, Lei Chen, Tao Sun, Yangbo Su, Yiming Feng, Jinqiang Ma, Huimin Liang, Chuansheng Zheng

**Affiliations:** ^1^Department of Radiology, Union Hospital, Tongji Medical College, Huazhong University of Science and Technology, Wuhan, China; ^2^Hubei Province Key Laboratory of Molecular Imaging, Wuhan, China

**Keywords:** percutaneous nephroscopy, kidney, hemorrhage, embolization, embolism experience

## Abstract

**Objective:** The purpose of this study was to evaluate the therapeutic efficacy and safety of superselective renal arterial embolization (SRAE) in the treatment of patients with renal hemorrhage after percutaneous nephroscopy (PCNL). In addition, embolization techniques and embolization materials during operation were also worthy of further discussion.

**Methods:** From February 2015 to December 2019, clinical data of 49 consecutive patients with renal hemorrhage after PCNL were retrospectively analyzed. Demographic and clinical data of patients were recorded, changes in serum creatinine values were analyzed, and the safety and efficacy of TAE were evaluated. Clinical experience was also recorded.

**Results:** A total of 49 patients underwent angiography, of which 46 patients received SRAE due to positive hemorrhagic foci detected by angiography, and the technical success rate of 46 patients was 100%. Among the three patients who did not receive embolization, one patient underwent nephrectomy, and two patients improved with conservative treatment, with a clinical success rate of 98%. There was no statistically significant difference between serum creatinine before PCNL and 7 days after SRAE (101.6 ± 36.5 to 100.5 ± 27.1 μmol/L; *P* = 0.634), and no significant change was observed in serum creatinine at the last follow-up (99.4 ± 34 μmol/L, *P* = 0.076). No major complications occurred after embolization.

**Conclusions:** SRAE is safe and effective in patients with renal hemorrhage after PCNL. The experience of interventional therapy and the choice of embolization materials in this study may provide certain benefits for the treatment of patients with renal hemorrhage after PCNL.

## Introduction

Percutaneous nephrolithotomy (PCNL) is a safe and effective treatment for patients with upper urinary tract stones, including large and complex urinary calculi (1, 2). Despite the increase of surgical experience and the improvement of surgical instruments, PCNL is still an invasive method with a certain incidence of complications. Hemorrhage is the most serious and dangerous complication of PCNL (3, 4). Although it is usually self-limited and can be controlled conservatively (5, 6), severe hemorrhage is still a troublesome problem, occurring in 0.3–4.7% of cases and requiring expeditious attention (1, 7, 8). In these patients, hemostasis by surgical exploration may lead to nephrectomy. As a minimally invasive interventional therapy, renal artery embolization is a mature method for the treatment of massive hemorrhage (9).

Since 1970's, percutaneous renal arterial embolization has been used to control renal bleeding (10). It has been reported that up to 20% of patients with hemorrhage after PCNL require transfusion, while <1% require angiographic embolization (5, 11–13). Recently, with the introduction of new interventional devices and embolization materials, embolization technology has been further developed (14). In order to minimize the damage of renal function, superselective renal arterial embolization (SRAE) has been widely performed (14–17).

In this study, we retrospectively evaluated the safety and efficacy of SRAE in the treatment of hemorrhage after PCNL. In addition, although renal artery embolization has been regarded as the treatment of choice for postoperative bleeding of PCNL (18–20), there are still reports of recurrent hemorrhage after embolization (1, 7), and even Mao et al. reported that up to 17.3% of patients experienced initial treatment failure and underwent repeat SRAE (16). The failure of embolization may be partly due to immature embolization techniques and improper selection of embolization materials. Therefore, we reported our experience in the clinical management of SRAE for the treatment of renal hemorrhage after PCNL.

## Materials and Methods

### Patients

The present retrospective study received local hospital ethic committee approval. Written informed consent was obtained from all patients prior to treatment.

In this cohort study at our institution, we retrospectively reviewed 49 consecutive patients with clinically active bleeding after PCNL who underwent angiography (Table 1) from February 2015 to December 2019. We investigated 41 males and eight females with an average age of 53.3 ± 9.9 (range, 31–69) years. All PCNLs were performed by four experienced urologists who were not involved in the retrospective study. The indications for angiography and SRAE included the following: (1) clinical manifestations of vascular injury such as hemodynamic instability, gross hematuria, abdominal pain, and/or hypovolemic shock; (2) uncontrolled intraoperative blood loss; (3) imaging evidence suggested active bleeding, such as pseudoaneurysm (PA), active contrast extravasation, arteriovenous fistula (AVF), and large hematoma.

**Table 1:** Basic characteristics of patients.

**Characteristic**	**No. patients (%)**
**Gender**	
Male	41 (83.7%)
Female	8 (16.3%)
**Age (years)**	
Mean ±*SD*	53.3 ± 9.9
Range	31–69
**Hypertension**	
Yes	19 (38.8%)
No	30 (61.2%)
**Diabetes mellitus**	
Yes	7 (14.3%)
No	42 (85.7%)
**Antiplatelet therapy**	
Yes	4 (8.2%)
No	45 (91.8%)
**Anticoagulation therapy**	
Yes	1 (2%)
No	48 (98%)
**Side**	
Right	24 (49.0%)
Left	25 (51%)
**Stone size (cm)**	
Mean ±*SD*	3.9 ± 1.7
Range	1.8–10.3
**Stone burden**	
Single	8 (16.3%)
Multiple	36 (73.5%)
Staghorn	5 (10.2%)
**Bleeding site**	
Upper pole	11 (23.9%)
Mid-pole	15 (32.6%)
Lower pole	17 (37.0%)
Mid-pole + Lower pole	2 (4.3%)
Upper pole + Mid-pole	1 (2.2%)
**Clinical characteristics**	
Reduced hemoglobin	29 (59.2%)
Gross hematuria	12 (24.5%)
Red fluid in the fistula or drainage bag	25 (51%)
Hypovolemic shock	4 (8.2%)
**Intervals between bleeding and the angiography (days)**	
Mean ±*SD*	4.7 ± 3.6
Range	0–14
**Hemoglobin decrease (g/dL)**	
Mean ±*SD*	4.3 ± 2.4
Range	1.1–10.7
**Transfusion requirement**	
Yes	28 (57.1%)
No	21 (42.9%)
**Units of transfusion**	
Mean ±*SD*	3.8 ± 1.9
Range	2–8

*SD, Standard deviation*.

### Angiography and Embolization Technique

**Figure 1:**
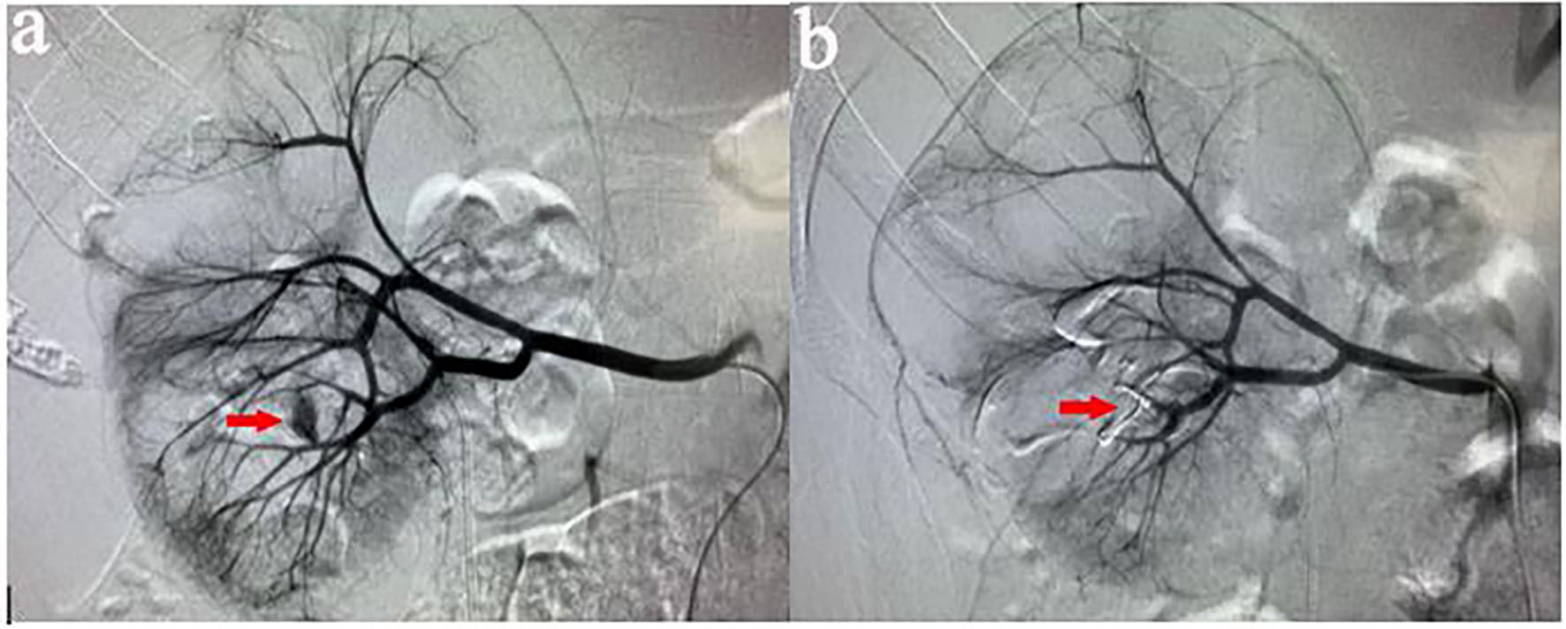
**(a)** A 37-years-old male patient developed gross hematuria after PCNL. Two days later, right renal arteriography revealed a contrast extravasation of the right inferior interlobular artery. **(b)** After the target vessel was found and embolized by coils, the angiography showed no extravasation of contrast agent.

**Figure 2:**
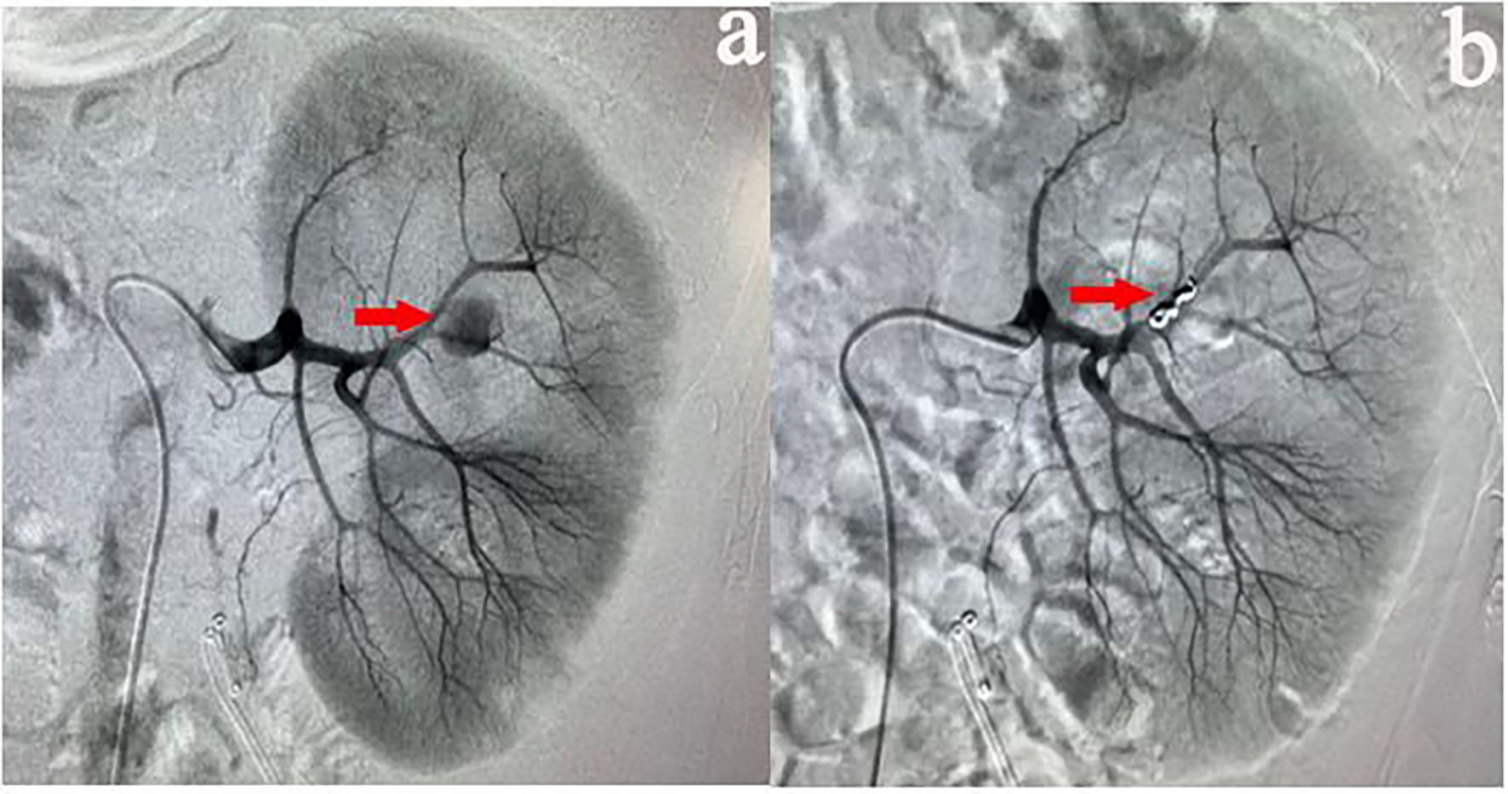
**(a)** A 35-years-old male patient showed a progressive decrease in hemoglobin after PCNL. Two days later, left renal arteriography revealed PA in the left superior lobular artery. **(b)** After the target vessel was found and embolized by coils, no PA was observed on the angiography.

**Figure 3:**
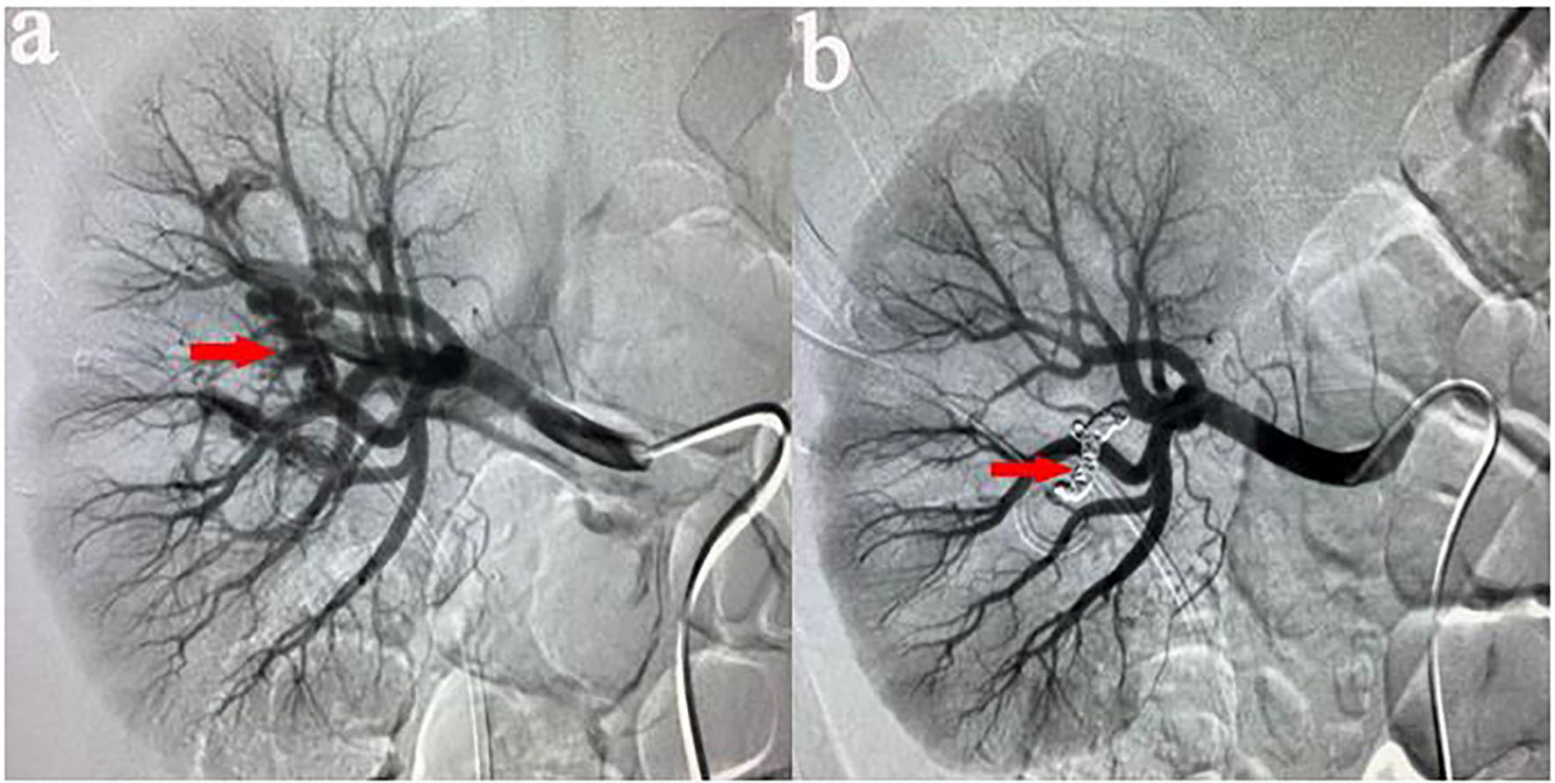
**(a)** Male, 46 years old, showed bloody fluid in the drainage bag after PCNL, and decreased hemoglobin. Eight days later, right renal arteriography revealed the presence of AVF in the right middle lobular artery. **(b)** After the target vessel was found and embolized by coils, AVF disappeared on angiography.

All procedures were performed by our experienced interventional radiologists in close consultation with the urologists in an emergency basis. Digital subtraction angiography was usually performed by puncture of the right femoral artery under local anesthesia using a modified Seldinger technique. Then a 5-F sheath (Terumo, Tokyo, Japan) was inserted. A 5-F pigtail, Cobra or Yashiro catheter (Cook, Bloomington, Indiana, USA) was first placed in the abdominal aorta and the opening of the renal artery on the healthy side for angiography, and the angiography performance of the healthy side of the kidney was observed. Renal arteries, accessory renal arteries, lumbar arteries, and other collateral vessels were also evaluated. The catheter was then placed at the opening of the renal artery on the affected side for renal arteriography to determine the cause of renal hemorrhage. In addition, imaging at a transverse or oblique angle can help determine the exact location of arterial damage. The overflow of contrast agent (Figure 1), the formation of PA (Figure 2), and AVF (Figure 3) were mainly observed.

Contrast extravasation is one of the common abnormality, overflow of contrast agent to flake gathered in the essence of the kidney and renal subcapsular. AVF is another angiographic manifestation, usually because an artery is directly connected to the renal vein through the damaged kidney tissue. During angiography, renal venous filling can be seen at the arterial stage. PA formation is an important manifestation of angiography. It is usually located at the end of the damaged artery and presents as a circular retention and aggregation of contrast agent.

The coaxial 2.8-F microcatheter (Terumo, Tokyo, Japan) was then guided by a 0.014-inch guide wire (Terumo, Tokyo, Japan) as close to the bleeding site as possible. Before embolization, angiography should be performed to ensure that the catheter is in the correct position. We selected different embolization materials based on whether the microcatheter was located in the target vessel and the angiographic results.

In order to minimize the loss of normal renal parenchyma, the microcatheter was selected into the culprit vessel as far as possible. In this case, the satisfactory effect could be obtained by embolization with appropriate coils (Cook, Bloomington, Indiana, USA). If, unfortunately, the microcatheter was not superselected into the targeted vessel, the embolization of gelatin sponge particles (300–500 μm, Cook, Bloomington, Indiana, USA) or PVA particles (300–500 μm, Cook, Bloomington, Indiana, USA) can be used first, followed by the coils. For AVF, the best treatment was to select the appropriate size of the coils according to the target vessel diameter. The microcatheter was superselected into the distal of PA, and then the coils were placed from the distal to the proximal of PA.

Reexamination angiography of the renal artery was performed to confirm complete occlusion of the culprit artery, to show patency of the remaining vessels, and to assess the percentage of renal parenchymal loss.

### Definition and Evaluation of Data

Technical success was defined as angiography showing complete embolization of the hemorrhage without any evidence of contrast extravasation. Clinical success was defined as the absence of further bleeding, improvement in the patient's clinical symptoms, and no need to repeat SRAE or surgical intervention. Complications were assessed using the Society of Interventional Radiology (21). Major complications were defined as events leading to death and disability that increase the level of care, or result in hospital admission, or substantially lengthen the hospital stay such as displacement of coils, loss of renal function, or renal artery dissection. All other complications were considered minor.

### Assessment and Follow-Up

Demographic, clinical and laboratory data were collected. To assess the effect of SRAE on renal function in patients with renal hemorrhage after PCNL and serum creatinine data within 7 days after embolization were evaluated. In addition, serum creatinine data from the last follow-up of patients were collected to assess the long-term effects of SRAE on renal function. The follow up time was 6–64 months, with an average of 32.1 months.

### Statistical Analysis

All analyses were performed using SPSS software (Version 24.0; IBM, Armonk, New York), and *P* < 0.05 was considered statistically significant. Discrete variables were represented as numbers with percentage, and quantitative data were presented as mean ± standard deviation (*SD*). Quantitative data were evaluated by Student's *t*-test.

## Results

### Study Population and Patient Characteristics

A total of 49 patients underwent angiography. All 49 patients presented abnormal clinical manifestations requiring angiography, including 29 (59.2%) patients with reduced hemoglobin, 12 patients (24.5%) with gross hematuria, 25 patients (51%) with red fluid in the fistula or drainage bag, and four patients (8.2%) with hypovolemic shock. The mean interval between bleeding and the angiography was 4.7 ± 3.6 days (range, 0–14 days). Of the 49 patients, 25 had left renal hemorrhage and 24 had right renal hemorrhage. Among them, there were 11, 15, and 17 patients with upper, middle and lower segment renal vascular injuries, in addition, two patients with middle and lower branches injury, and one patient with middle and upper branches injury. After PCNL and before angiography, the average Hb decrease was 4.3 ± 2.4 g/dL (range, 1.1–10.7 g/dL). In this study, 28 (57.1%) patients received transfusion with an average volume of 3.8 units.

### Angiography and Embolization Materials

In this study, all 49 patients underwent angiography, bleeding foci were observed in 93.9% (46/49) of the patients. Angiographic findings were summarized in Table 2, including contrast extravasation (32.7%, 16/49), PA (20.4%, 10/49), PA with AVF (20.4%, 10/49), contrast extravasation with AVF (12.2%, 6/49), and AVF (8.2%, 4/49). Meanwhile, no bleeding foci were found in three patients (6.1%, 3/49) by angiography. Of the 46 patients who received SRAE, 25 (54.3%) were treated with coils, 11 (23.9%) were treated with PVA combined coils, and 10 (21.7%) were treated with gelatin sponge particles combined coils (Table 2). The average number of coils used was 2.6 (range, 1–7 coils).

**Table 2:** Angiographic findings and embolization materials.

**Characteristic**	**Number (%)**
**Angiographic findings**	
Contrast extravasation	16 (32.7%)
PA	10 (20.4%)
PA & AVF	10 (20.4%)
Contrast extravasation & AVF	6 (12.2%)
AVF	4 (8.2%)
No bleeding foci	3 (6.1%)
**Embolic material used**	
Coils	25 (54.3%)
Colis & PVA	11 (23.9%)
Coils & GS	10 (21.7%)

*PA, Pseudoaneurysm; AVF, Arteriovenous fistula; GS, Gelatin sponge*.

### Technical and Clinical Outcomes

All the 49 patients underwent angiography, among which 46 patients with positive angiographic findings successfully underwent embolization of target vessels, with a technical success rate of 100%, while the remaining three patients failed to carry out further operation due to the absence of bleeding foci. The bleeding vessels in this study were all branches of renal artery. Among the three patients with negative angiographic findings, 1 patient needed nephrectomy to save his life and had no fresh hemorrhage after nephrectomy, and the other two patients were probably due to venous bleeding, which gradually stopped with conservative treatment. The clinical performance of 46 patients who were successfully treated with SRAE gradually improved, and no transfusion was performed after embolization. Among the three patients who did not receive embolization, one patient underwent nephrectomy and two patients improved with conservative treatment, with a clinical success rate of 98%.

### Renal Function and Complications

**Figure 4:**
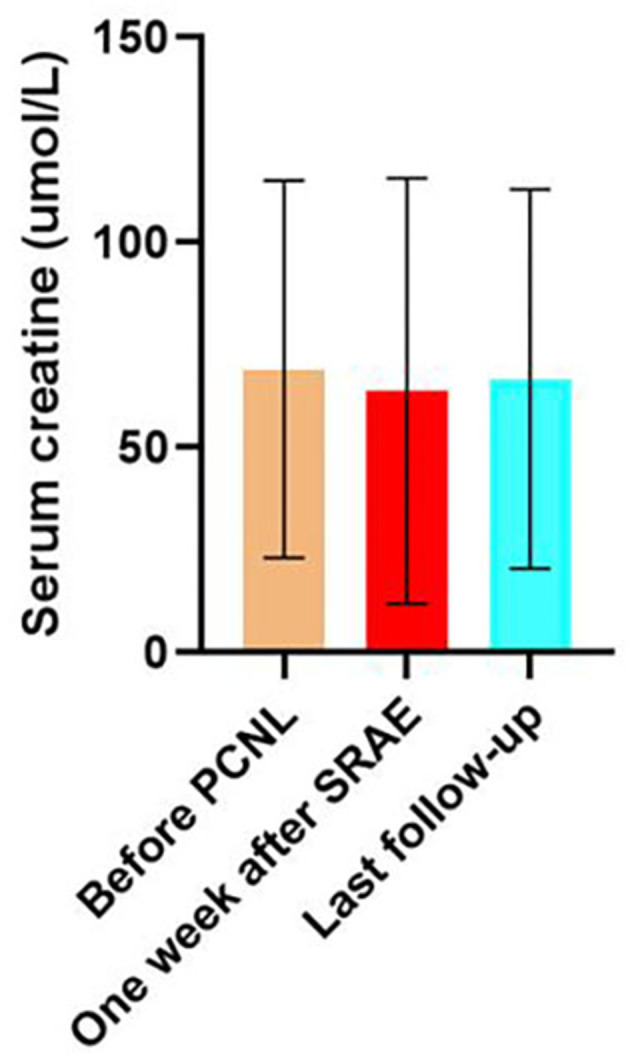
The histogram showed no significant difference in serum creatinine at 7 days after SRAE and at the last follow-up compared with that before PCNL.

**Figure 5:**
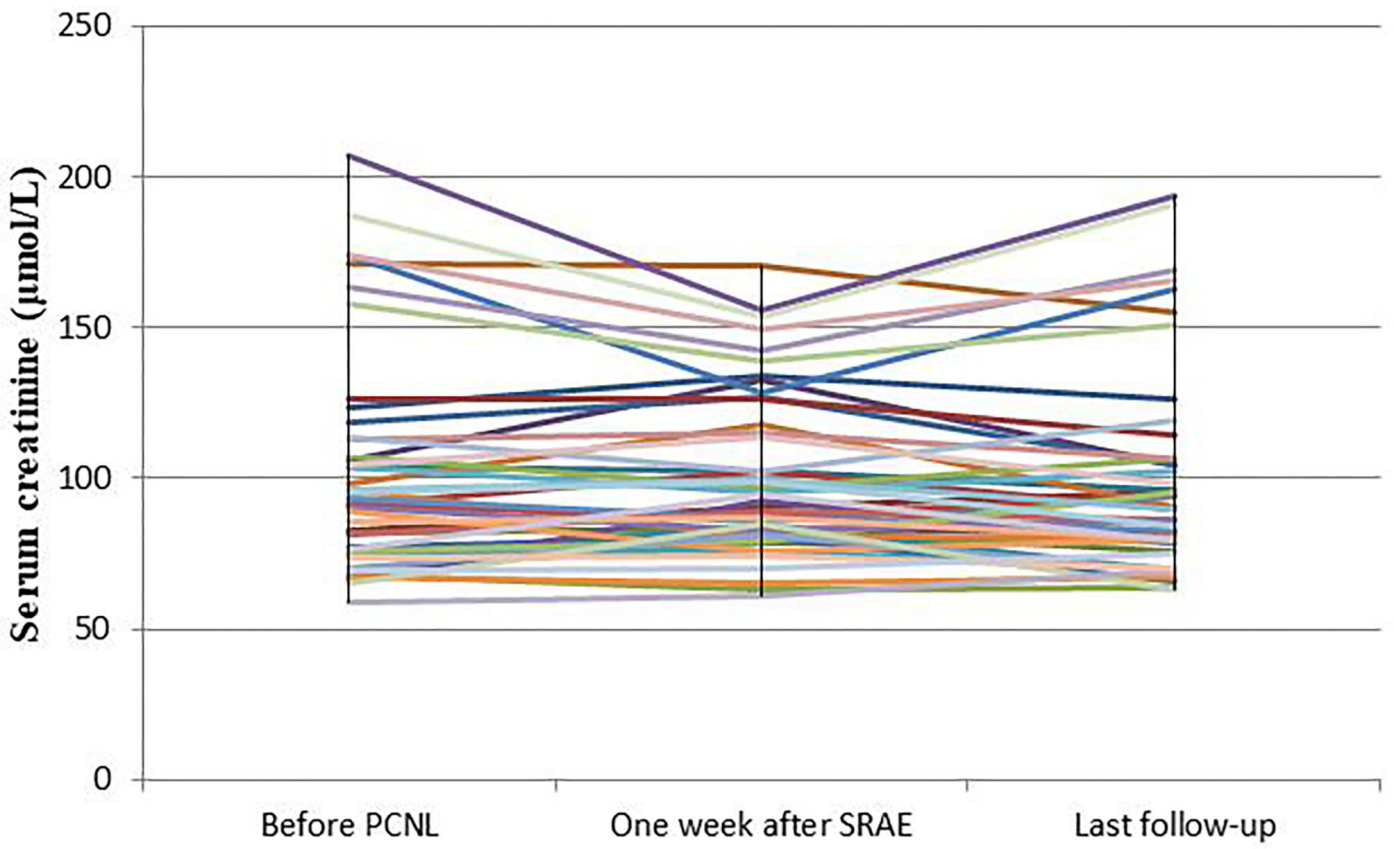
The curve showed the creatinine changes before and after embolization in 46 patients who received SRAE.

The mean serum creatinine of patients before PCNL was 101.6 ± 36.5 μmol/L, and that of patients 7 days after SRAE was 100.5 ± 27.1 μmol/L (*P* = 0.634). The mean serum creatinine of the patients at the last follow-up was 99.4 ± 34 μmol/L, and there was no statistical difference compared with that before SRAE (*P* = 0.076) (Figure 4). Meanwhile, Figure 5 showed the creatinine changes before and after embolization in 46 patients who received SRAE. In this study, a total of 46 patients with postoperative PCNL bleeding received SRAE, and no major complications (coil displacement, renal artery dissection, and loss of renal function) occurred after embolization. Common minor complications occurred in 25 patients (54.3%), including 19 patients (41.3%) with fever, 16 patients (34.8%) with flank pain, two patients (4.3%) with access site hematoma, These symptoms lasted 2–7 days and were relieved by symptomatic treatment.

## Discussion

Currently, SRAE has become a recognized treatment for severe and persistent renal bleeding after PCNL that fails to achieve a satisfactory outcome through conservative treatment (1, 19). The technical and clinical success rates of embolization for renal hemorrhage have been reported to be 87–100% and 57–100%, respectively (18). In this study, 46 patients with positive angiographic findings underwent SRAE and underwent renal artery angiography again after embolization, showing complete occlusion of the target artery, with a technical success rate of 100%. Meanwhile, the clinical success rate of this study was also 100%, which was significantly better than previous studies (5, 7, 18).

Careful and meticulous angiography and reasonable selection of embolization materials based on the results of angiography may explain the high technical and clinical success rates in the present study. In addition to renal arteries, accessory renal artery, lumbar arteries and other collateral arteries should be observed, and local magnification or rotation at a certain angle should be performed when necessary during angiography. El-Nahas et al. (15, 22) used only platinum microcoils to embolize the injured vessels, Zeng et al. (7) used gelatin sponges only for certain small and individual PAs. However, study has shown that the use of gelatin sponges alone is a risk factor for bleeding after embolization (7). Similarly, another study (1) embolized a subset of patients with gelatin sponges and found that five patients (13.4%) needed embolization again for recanalization of the embolized vessel. In this study, we did not embolize the target vessels with gelatin sponges alone. At the same time, the balance between thorough hemostasis and maximum protection of the patient's kidney was considered during embolization.

It is reported that the average time interval from bleeding to angiography was 1–5 days (9, 18, 20), and the average time interval in this study was 4.7 days. For some patients, early angiography may be able to detect fatal bleeding and reduce transfusion and complications. Studies (3, 4, 11) have shown that stones with large diameter, excessive puncture times during PCNL, incorrect renal puncture and relatively large nephroscope passage are prognostic factors for severe renal hemorrhage. Therefore, for such patients with gross hematuria and decreased hemoglobin after PCNL, early angiography can benefit these patients more. These patients were not analyzed in this study, which warrants further study.

Similar to other studies (18, 19, 23), contrast extravasation was the most common angiographic manifestation in this study, followed by PA and PA combined with AVF, while AVF or AVF combined with contrast extravasation was also common. Negative angiographic findings were also present in our study. This may be due to a slight tear of vessels, slow rate of bleeding (<0.5 ml/min), bleeding vasospasm, or bleeding from vein injuries (20). For patients with no hemorrhagic foci found during angiography, a study using empirical embolization also achieved satisfactory results (18). In our experience, we mainly performed empiric embolization based on the puncture site of PCNL and the location of perirenal hematoma on imaging. Due to the poor renal function and the absence of perirenal hematoma, in order to avoid further damaging the renal function of the patients, none of the three patients in this study underwent empiric embolism after careful evaluation. Two of the patients stopped bleeding after conservative treatment, and one patient eventually underwent a life-saving nephrectomy.

Percutaneous renal artery embolization is a minimally invasive treatment with little effect on renal function (15). Especially after the application of coaxial catheter, subsegment vascular catheterization and more distal embolization are possible, and the catheter can enter into the target vessel more accurately, so as to avoid peripheral renal parenchymal injury (17). The safety of SRAE in the treatment of renal hemorrhage has been confirmed in many studies (9, 18, 20). In the present study, there was no statistical difference in serum creatinine values before the percutaneous renal procedure and those measured 7 days after TAE (*P* = 0.634). Long-term follow-up showed no significant change in renal functions.

However, SRAE is not without complications. Postembolization syndrome is the most common complication, and Somani et al. (24) reported that 50% of patients had this syndrome. In our series, 19 patients (41.3%) had fever, 16 patients (34.8%) had flank pain. This is mainly due to renal tissue ischemia and necrosis caused by partial renal parenchyma devascularization after target vessel embolization. Careful preoperative planning and attention to important technical aspects of SRAE ensured that no serious complications occurred in this study.

Retrospective nature is the main limitation of this study, which leads to a certain selection biases. Due to the continuous development of interventional techniques and embolization materials, considering the era effect, this study only reviewed the clinical data of patients who received SRAE treatment for renal hemorrhage after PCNL surgery in the last 5 years.

In conclusion, SRAE is safe and effective in the treatment of renal hemorrhage after PCNL. Although SRAE has been recognized as the standard method for severe bleeding after PCNL, there is still no standard consensus on the method of interventional treatment and the choice of embolization materials. Therefore, the experience of our center may be helpful to some extent.

## Data Availability Statement

The datasets used in this study are available from the corresponding author upon reasonable request.

## Ethics Statement

The studies involving human participants were reviewed and approved by Union Hospital, Tongji Medical College, Huazhong University of Science and Technology. The patients/participants provided their written informed consent to participate in this study.

## Author Contributions

XD, YR, PH, LC, and TS collected the patients' data. XD drafted the manuscript. XD, YR, HL, and CZ revised the manuscript, made substantial contributions to the design of the work, and have revised the manuscript substantively. LC, TS, YS, YF, and JM analyzed and interpreted the data. HL and CZ made substantial contributions to the conception of the work. All authors read and approved the final manuscript.

## Conflict of Interest

The authors declare that the research was conducted in the absence of any commercial or financial relationships that could be construed as a potential conflict of interest.

## Abbreviations

PCNL: percutaneous nephroscopy
SRAE: superselective renal arterial embolization
PA: pseudoaneurysm
AVF: arteriovenous fistula
SD: standard deviation.
